# Sheet Protector Strategy for Western Blot to Reduce Antibody Consumption and Incubation Time

**DOI:** 10.1186/s12575-025-00300-6

**Published:** 2025-09-24

**Authors:** Sunmin Kwon, So Yeong Lee, Gun Kim

**Affiliations:** College of Veterinary Medicine and Research Institute of Veterinary Science Laboratory of Veterinary Pharmacology, Seoul National University, 08826 Seoul, Republic of Korea

**Keywords:** Western blotting, Antibodies, Antibody conservation, Immunoblot, Immunodetection, Protein, Sheet protector

## Abstract

**Background:**

Western blot is one of the most routinely conducted biochemical assays due to its technical ease and relatively low cost. The use of antibody is at the center of Western blot assay, providing great sensitivity and specificity. However, challenges can be posed when using a rare antibody stock. There have been efforts to improve the Western blot procedure to minimize the use of antibody, but these methods require specialized devices.

**Results:**

In this study, we hypothesized that the conventional large pool of antibody is not essential for detection and attempted applying only a small volume of antibody. We used sheet protector (SP), a common stationery material that protects office documents, to effectively distribute the antibody solution over the nitrocellulose (NC) membrane. This way, 20–150 µL antibody solution was sufficient for mini-sized membrane, which was adjustable depending on the size of the membrane. We confirmed that the sensitivity and specificity of this SP strategy was comparable to conventional (CV) method. The SP strategy brought a few additional advantages including: (1) antibody incubation without agitation (2), incubation at room temperature, and (3) faster detection on the order of minutes. Finally, we examined 15-min incubation SP protocol using time-series apoptosis samples.

**Conclusions:**

We propose that SP strategy is a universally accessible approach using a common consumable, greatly enhancing the efficiency of antibody consumption and incubation time in Western blot assays.

**Supplementary Information:**

The online version contains supplementary material available at 10.1186/s12575-025-00300-6.

## Background

Western blot assay is an indispensable technique for the detection and quantification of proteins. Since its introduction 44 years ago, it remains an essential technique for protein analysis [1], being the most frequently used protein-related technique in PubMed-listed publications [2]. Western blot assay relies heavily on antibodies for the sensitivity and the specificity of the detection of the target proteins in a complex biological sample. Therefore, the quality and reliability of antibodies are crucial in Western blot assay [3, 4]. Antibodies are costly consumables, and studies using rare or expensive antibody stocks often face limitations. In addition, batch-to-batch variability in antibody production raises concerns about the reliability of results [3, 4]. These issues have long been recognized, and several researchers have attempted to reduce antibody consumption [5–7]. However, these approaches are not as practical due to the need for specialized equipment. Therefore, a significant challenge is posed in saving antibody stocks.

Classically, the Western blot assay involves an incubation process where the membrane is put in a container with the antibody solution [8]. The container is placed on a rocker or shaker for an efficient reaction between the antibody and the target proteins. Though it may vary among laboratory setups, 10-mL antibody solution at working concentration is typically required to fully cover the protein-transferred membrane.

The current practice of antigen probing in Western blot assays relies on large volume (bulk) antibody solution to probe the antigens. However, since the antigens are immobilized on the surface of the membrane, the binding reaction occurs at the interface of the membrane and the antibody solution. As the reaction proceeds, the local antibody near the membrane depletes, and is replenished from the bulk reservoir by diffusion. The large antibody pool maintains constant antibody concentration throughout the reaction.

Questions arise as most of the antibody in the bulk reservoir remains unreacted and will be eventually discarded. Since this practice wastes a substantial amount of antibody, some researchers attempt to reuse antibodies, with the risk of microbial contamination or antibody degradation. In addition, maintaining constant antibody concentration during the reaction might not be necessary, as the nature of the antibody-antigen binding is near-irreversible due to the extremely slow dissociation kinetics of antigen-antibody complex. Therefore, we explored strategies to probe the antigen with minimal antibody consumption.

## Methods

### Preparation of Cell Lysate Sample for Western Blot

A HeLa cell line (Korean Cell Line Bank, 10002) was cultured using high-glucose DMEM medium (WELGENE, LM 001–05) containing 10% FBS (S001-07) and 1% antibiotic/antimycotic solution (LS 203-01). The cells were harvested using a trypsin buffer (LS 015 − 08) and lysed with RIPA buffer (Thermo Fisher Scientific, 89900). The protein concentration of the lysate was determined by BCA assay kit (23225). Western blot sample was made by adding PAGEST^™^ (GeneAll, 751-001), adjusting the final protein concentration to 1 mg/mL. The samples were serially diluted and stored in a deep freezer at −70 °C.

### Gel Electrophoresis and Protein Transfer

Gel electrophoresis was performed as previously described [8]. 8%, 10%, or 12% acrylamide gel was made with 30% acrylamide/bis solution (Bio-Rad, 1610156), 1.5 M Tris-HCl, pH 8.8 (Biosesang, TR4020-050-88), 1 M Tris-HCl, pH 6.8 (TR2016-050-68), Sodium dodecyl sulfate (S1010), TEMED (Sigma-Aldrich, T9281), and ammonium persulfate (A3678). HeLa cell lysate samples (10 µg per well) were loaded into each well, along with Protein size marker ExcelBand™ (SMOBIO, PM2700). Electrophoresis was performed using the PowerPac™ HC High-Current Power Supply (Bio-Rad, 1645052). The resulting gel was transferred onto AmershamTM ProtranTM 0.2 μm NC membrane (Cytiva, 1060001). The protein transfer to the NC membranes was confirmed using Ponceau S staining (Merck, P7170).

### Washing

Membranes were washed three times with tris-buffered saline with Tween 20 (TBST) at 200 revolutions per min (RPM) on an orbital shaker (DAIHAN, SHO-1D) for 5 min per wash. TBST was made by adding 0.1% Tween-20 (Biosesang, T1027) to the TBS buffer which consists of 10 mM Tris (T1016) and 150 mM NaCl (Sigma-Aldrich, S9888) in distilled water, titrated with 37% HCl (258148) to adjust the pH to 7.6.

### Blocking

Membranes were blocked using 5% skim milk solution for 1 h with gentle rocking. The 5% skim milk solution was prepared by adding skim milk (Becton Dickinson, DF.232100) to TBST at 5% (v/v).

### Antibody Probing

Working solution of primary antibody was prepared by diluting the antibody stock to a desired concentration with 5% skim milk solution. All antibodies were freshly prepared on the day of the experiment. In CV method, the skim-milk blocked membrane was placed in a container of proper size with 10 mL primary antibody working solution. The container was then placed on an orbital shaker inside a fridge (4 °C) to be incubated overnight (18 h), with agitation at 60 RPM on an orbital shaker (FINEPCR, SH30). In SP strategy, the skim-milk blocked membranes were transiently immersed into TBST to wash away excessive skim milk. The membrane was then thoroughly blotted using a paper towel to absorb any residual moisture. The semi-dried membrane was placed on a leaflet of a cropped sheet protector and a small volume of the primary antibody working solution was applied to the membrane. The upper leaflet of the sheet protector was gently placed on the membrane, allowing the antibody solution to disperse over the membrane as a thin liquid layer by surface tension. The SP, membrane, and antibody solution together constituted an SP unit. The SP unit was then incubated at different conditions according to the experimental scheme. When incubation over 2 h was necessary, the SP unit was placed on a wet paper towel and sealed inside a zipper back to prevent evaporation of antibody solution. After the incubation with primary antibody, the membrane was washed three times, and was incubated with horseradish peroxidase (HRP)-conjugated secondary antibody (GenDEPOT, SA001 and SA002) in a container for 1 h with gentle agitation on a rocker at RT.

### Detection and Densitometric Analysis of Protein Bands

The membrane was treated with WesternBright™ Quantum™ chemiluminescent substrate (Advansta, K-12045-D50) to produce HRP signal, which was measured using ImageQuant^™^ LAS-4000 mini (GE Healthcare). The molecular size and the signal intensity were analyzed using FIJI software [9].

### Pearson’s Correlation Analysis

GraphPad Prism 8.0 was used to calculate Pearson’s *r* and p-value. The combined *r* was calculated by first applying the Fisher Z-transformation to each individual *r*, averaging the resulting values, and then applying the inverse transformation to obtain the final combined *r*. The combined *p* was calculated by applying Fisher’s combined probability test.

## Results

### Formation of Minimal-Volume Antibody Layer by Sheet Protector

In our attempt to apply antibody at a minimal volume, the first challenge was how to evenly distribute antibody solution on the nitrocellulose (NC) membrane. The antibody-containing solution, TBST, did not effectively spread throughout the membrane due to its surface tension. We effectively distributed the solution over the membrane by overlaying a leaflet of a sheet protector (SP) on the solution (Figure S1 and Video S1). The solution maintained a certain thickness, balancing between the downward pressure from the weight of the SP leaflet and the counteracting pressure by surface tension of the solution. We empirically estimated the volume (µL) required to cover a 4.5 cm-long NC membrane (pore size 0.2 μm), $\:{V}_{cover}$, which was determined as $\:{V}_{cover}=10n$, where $\:n$ is the total lane number for 15-well comb. The $\:{V}_{cover}$ ranges 20–150 µL depending on the size of the experimental groups (Examples of $\:{V}_{cover}$ are presented in Table S1).

### Determination of Antibody Concentration for SP Strategy

In the SP strategy, the lack of large antibody pool can result in a decrease in antibody concentration, possibly leading to less efficient binding reactions. We investigated the relationship between the antibody concentration and the signal intensity to find the antibody concentration in SP strategy that produces similar signal intensity to its CV counterpart. Three housekeeping proteins (GAPDH, α-tubulin, and β-actin) were used since they are relatively abundant in mammalian cell lysate and are frequently used as loading controls.

We transferred a lane of HeLa cell lysate to NC membrane. For CV group, the membranes were incubated with 0.1 µg/mL antibody solution (10 mL) at 4 °C and were agitated overnight (18 h). For SP groups, antibody solutions (20 µL) at 0.1, 0.2, 0.5, and 1.0 µg/mL were applied to each membrane using SP strategy. Each SP-enclosed membrane was placed on a wet paper towel and sealed into a zipper bag to prevent it from drying up. They were incubated overnight at 4 °C, agitated by an orbital shaker. Then, we took the membrane out of the SP and followed the usual Western blot protocol for the rest of the assay. Overall, a tendency of positive correlation between the antibody concentration and the signal intensity was observed (Figure S2A–B and Table S2). We also found that SP strategy at 1.0 µg/mL antibody and CV method at 0.1 µg/mL produced signals of comparable intensity (Figure S2C). Therefore, we used antibodies for CV and SP at 0.1 and 1.0 µg/mL, respectively, in the following experiments.

### The Sensitivity and Specificity of the SP Strategy

**Fig. 1:**
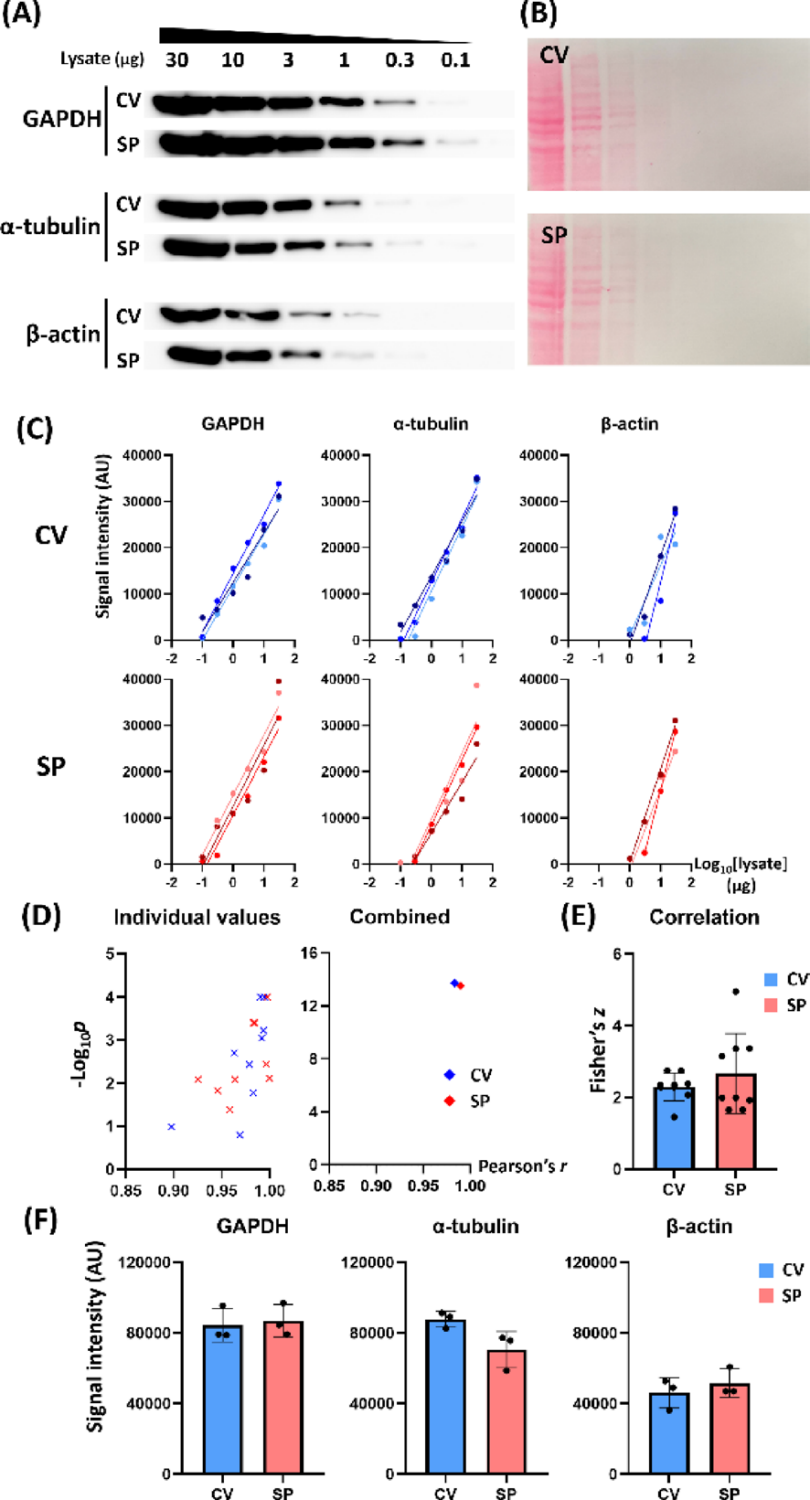
Sensitivity of SP strategy. (**A**) Representative Western blot images showing semi-quantitative detection of target protein by CV and SP. (**B**) Ponceau S-stained membranes used for CV or SP. (**C**) The signal intensity against log_10_[lysate] exhibits linear correlation for each membrane set in both CV and SP. The lower limit of detection was 0.1 µg (GAPDH), 0.3 µg (α-tubulin), and 1 µg (β-actin). (**D**) The -log_10_*p* values were pooled across the three antibody groups and plotted against Pearson’s *r* values (left). Combined *r* and *p* values for these data were obtained using Fisher Z-transformation and Fisher’s combined probability test, respectively (right, *n* = 9). (**E**) Fisher’s *z* from the pooled Pearson’s *r* values between CV and SP exhibited no significant difference. (**F**) CV and SP target band intensity summed across 30–1 µg lysates in each antibody exhibited no significant difference

Semi-quantitative detection is one of the key features of the Western blot assay. To confirm semi-quantitative detection power of SP strategy and to evaluate its dynamic detection range, we loaded 0.1–30 µg HeLa cell lysates and probed them using CV (0.1 µg/mL) and SP (1.0 µg/mL) methods, respectively (Fig. 1A–B). Both the CV and SP methods showed a robust linearity between the log_10_[amount of loaded lysate] and the detected signal intensity in all three tested antibodies (Fig. 1C and Table S3). The lower limit of the linear detection range was 0.1 µg, 0.3 µg, and 1 µg for GAPDH, α-tubulin, and β-actin, respectively.

We pooled the independent datasets of the three antibodies and plotted -log_10_[p-value] against Pearson’s *r* (Fig. 1D). We then calculated the combined *r* and *p* using Fisher Z-transformation and Fisher’s combined probability test, respectively (Fig. 1D). The combined parameters in both the CV (*r* = 0.9830 and -log_10_*p* = 13.7132) and the SP (*r* = 0.9895 and -log_10_*p* = 13.5167) groups exhibited significant linear relationships. The Fisher’s *z* for the CV (mean *z* = 2.3807) and SP (mean *z* = 2.6243) groups were not significantly different (Fig. 1E, Mann-Whitney test, *n* = 9). In addition, the summed band intensities for 1–30 µg lysates across the antibodies did not differ statistically between the CV and SP groups (Fig. 1F, Mann-Whitney test, *n* = 3).

**Fig. 2:**
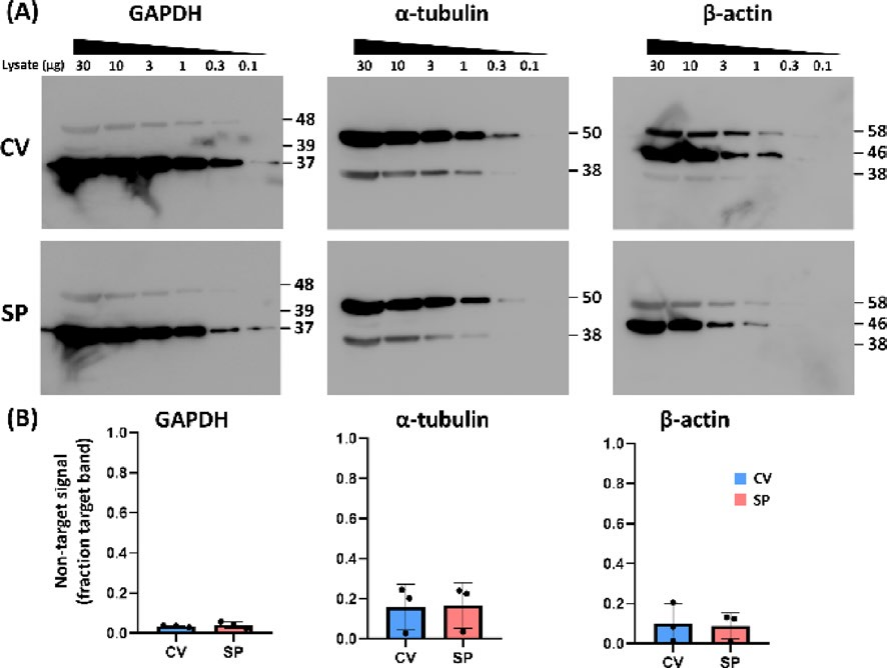
Specificity of SP strategy. (**A**) Representative whole blot image of Fig. 3A showing additional bands of non-theoretical molecular weight in CV and SP methods. (**B**) Non-target intensity relative to target (summed across 30 and 10 µg lysates) in CV and SP showed no significant difference

Next, we investigated whether SP procedure increases the nonspecific signals compared to CV method. The specificity of the SP strategy was examined using the results in Fig. 1A, analyzing the target bands and non-target bands. The detection pattern and the band size of non-target signals of each antibody were identical between the CV and SP groups (Fig. 2A). We measured and summed the signal intensities of the 10 and 30 µg lysate for the target bands and non-target bands, respectively. The relative non-target signal was expressed as a fraction of the target band signal, which was not significantly different between the CV and SP groups in all three antibodies (Fig. 2B, Mann-Whitney test, *n* = 3).

In summary, the SP strategy showed a robust lysate-signal relationship of the target band as did the CV method, demonstrating the semi-quantitative detection of the SP strategy. In addition, the extent of non-target signals was on a similar level in both CV and SP methods. These results indicate that employing SP strategy at an optimized antibody concentration does not compromise the sensitivity and specificity of Western blot assays.

### Incubation without Agitation

**Fig. 3:**
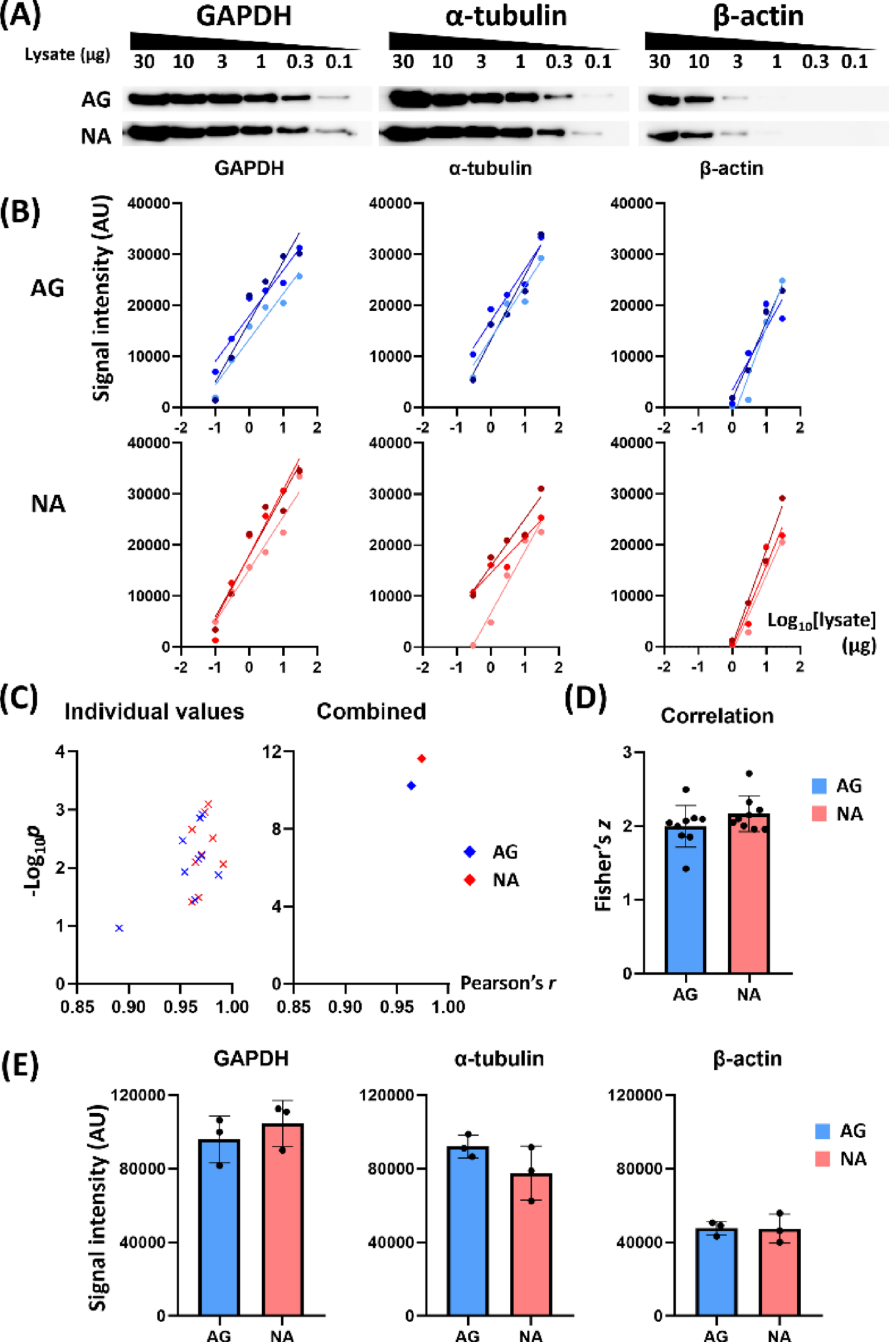
Agitation is not necessary in SP strategy. Antibodies were applied to NC membrane using SP and were incubated overnight at 4 °C with or without agitation. The signals of agitated (AG) and non-agitated (NA) incubation were detected side-by-side for accurate comparison. (**A**) Representative Western blot images comparing AG and NA. (**B**) The signal intensity against log_10_[lysate] exhibits correlation for each membrane set in both AG and NA. The lower limit of detection was 0.1 µg (GAPDH), 0.3 µg (α-tubulin), and 1 µg (β-actin). (**C**) The -log_10_*p* values were pooled across the antibody groups and plotted against Pearson’s *r* values (left). Combined *r* and *p* values for these data were obtained using Fisher Z-transformation and Fisher’s combined probability test, respectively (right). (**D**) Fisher’s *z* from the pooled Pearson’s *r* values were compared between AG and NA groups exhibited no significant difference. (**E**) Target band intensity summed across 30–1 µg lysates in each antibody exhibited no significant difference between AG and NA

In CV method, agitation is a critical step in antibody incubation to facilitate the bulk movement of antibody solution for proper detection [8, 10, 11]. We speculated that agitation might not be as effective in the SP strategy due to the small liquid volume and could be omitted. We followed SP protocol with and without agitation during the 18-hour antibody incubation at 4 °C. The agitated (AG) and non-agitated (NA) groups exhibited similar band patterns and detection range (Fig. 3A), both showing semi-quantitative detection power with a highly linear relationship between the band signal intensity and the log_10_[lysate] (Fig. 3B and Table S4).

The combined Pearson’s *r* and combined *p* were calculated by pooling the datasets across the antibody groups (Fig. 3C). Both the AG (*r* = 0.9638 and -log_10_*p* = 10.2317) and NA (*r* = 0.9741 and -log_10_*p* = 11.6336) groups exhibited a strong correlation (Fig. 3C). The Fisher’s *z* in the AG and NA groups did not differ significantly (Fig. 3D, Mann-Whitney test, *n* = 9). Densitometric analysis showed that the sum of 1–30 µg band intensities of the AG and NA groups were not significantly different (Fig. 3E, Mann-Whitney test, *n* = 3).

### The 15-min protocol

We speculated that the removal of pre-filled TBST could facilitate the access of the antibody solution to the pore space, resulting in faster reaction. In addition, antibody incubation at room temperature (RT) is a valid option in SP strategy, since the antibody is not expected to be reused. Therefore, we hypothesized that the SP strategy can reduce the incubation time.

**Fig. 4:**
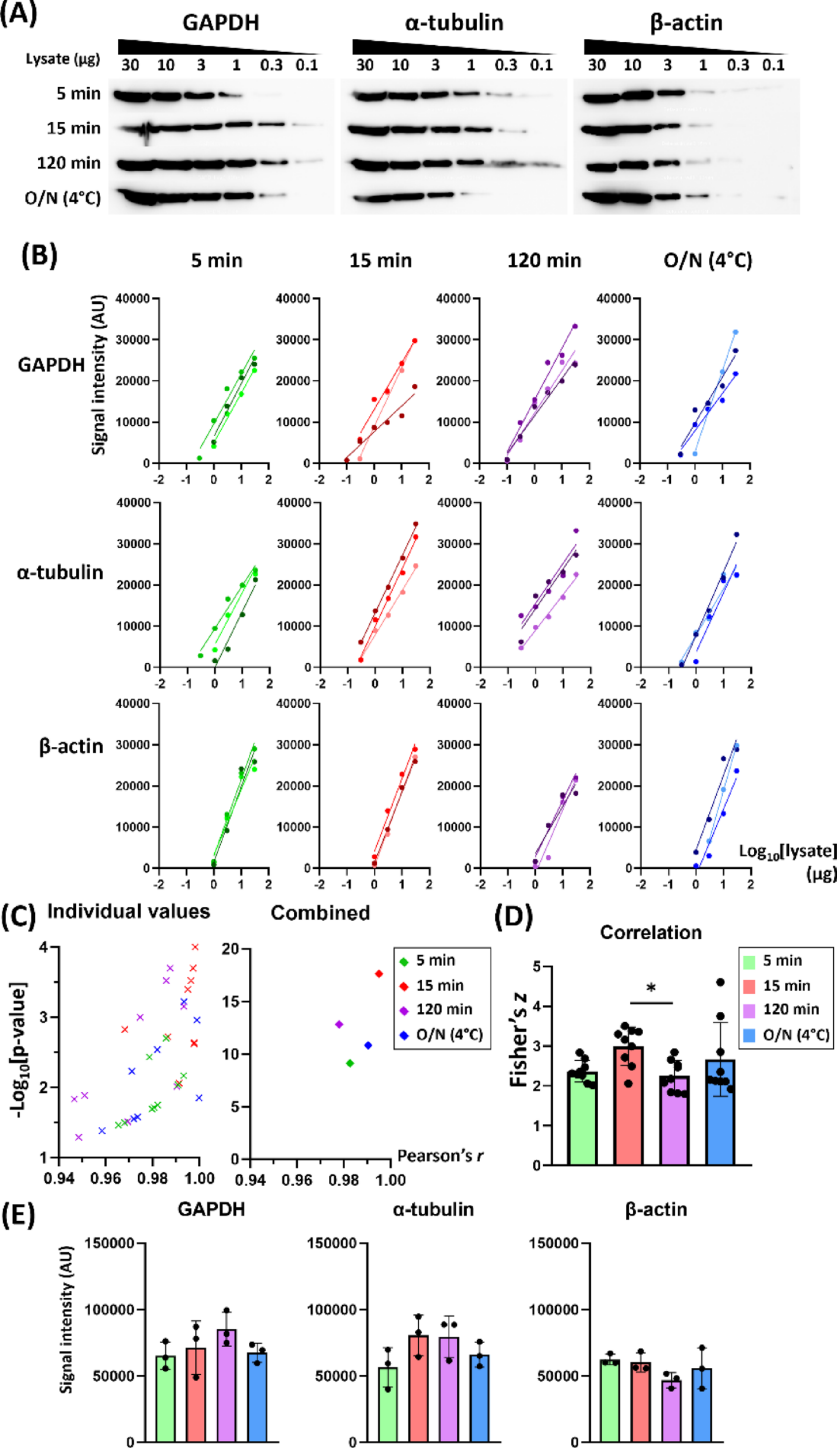
Comparison of semi-quantitative detection by incubation time. The membranes were incubated with antibody using SP strategy, without agitation, for a different duration. Incubation was conducted at room temperature except for overnight (O/N) group (4 °C). (**A**) Representative Western blot images comparing incubation time (5 min, 15 min, 120 min, and overnight) in SP. (**B**) The signal intensity against log_10_[lysate] exhibits linear correlation for each membrane set in all incubation groups. The lower limit of detection was 0.1 µg (GAPDH), 0.3 µg (α-tubulin), and 1 µg (β-actin). (**C**) The -log_10_*p* values were pooled across the antibody groups and plotted against Pearson’s *r* values (left). Combined *r* and *p* values for these data were obtained using Fisher Z-transformation and Fisher’s combined probability test, respectively (right). (**D**) Fisher’s *z* from the pooled Pearson’s *r* values were compared among 5 min, 15 min, 120 min, and O/N (4 °C) groups exhibited a difference between 15 min and 120 min groups. (**E**) Target band intensity summed across 30–1 µg lysate in each antibody exhibited no significant difference among the incubation time groups

We conducted Western blot assays using SP strategy with different incubation conditions: 5 min, 15 min, and 120 min groups at RT, and an 18-hour overnight group at 4 °C. All incubations were conducted without agitation. All groups produced bands with substantial signal intensities (Fig. 4A and Table S5). On visual inspection, the detection range tended to be narrower in the 5 min group for GAPDH and the overnight (4 °C) group in α-tubulin. However, we could find a robust correlation between the band intensity and log_10_[lysate] (Fig. 4B).

The datasets were pooled across the antibodies to plot Pearson’s *r* and -log_10_*p* (Fig. 4C). The combined Pearson’s *r* and the combined *p* for each group were as follows (Fig. 4D): 5 min (*r* = 0.9825 and -log_10_*p* = 9.1227), 15 min (*r* = 0.9950 and -log_10_*p* = 17.6211), 120 min (*r* = 0.9779 and -log_10_*p* = 12.8186), and overnight (*r* = 0.9904 and -log_10_*p* = 10.8350). The Fisher’s *z* values were different among groups (*p* = 0.0361, Kruskal-Wallis test, *n* = 9), where only 15 min and 120 min groups exhibited a statistical difference (Fig. 4D, *p* = 0.0355, Dunn’s multiple comparisons test). A densitometric sum of 1–30 µg lysates did not differ significantly among the groups in any of the antibodies (Fig. 4E, Kruskal-Wallis test, *n* = 3). Of note, overnight (4 °C) group, despite the longest incubation, did not produce a stronger signal than other groups.

These results indicate that the detective power of the SP strategy is not critically dependent on the antibody incubation time within the range of 5–120 min (RT) and overnight (4 °C) conditions. Each condition produced substantial signal with a robust linearity between the signal intensity and log_10_[lysate]. If we incubate the membrane for 15 min, it is 70-fold more time-efficient than the traditional overnight (1,080 min) protocol. More importantly, the overall timespan for Western blot assay which used to take two days can now be completed in a single day.

### Application of the Standard SP Protocol in Apoptosis Detection

We investigated whether the suggested SP protocol for Western blot can produce sufficient signals in diverse antibodies, significantly reducing both the antibody consumption and the assay time. We performed an apoptosis experiment on HeLa cells with 1 µM staurosporine, a well-known apoptosis inducer [12], and obtained the cell lysates at 0, 2, 4, 6, and 8 h post treatment and a negative control (0.1% DMSO).

**Fig. 5:**
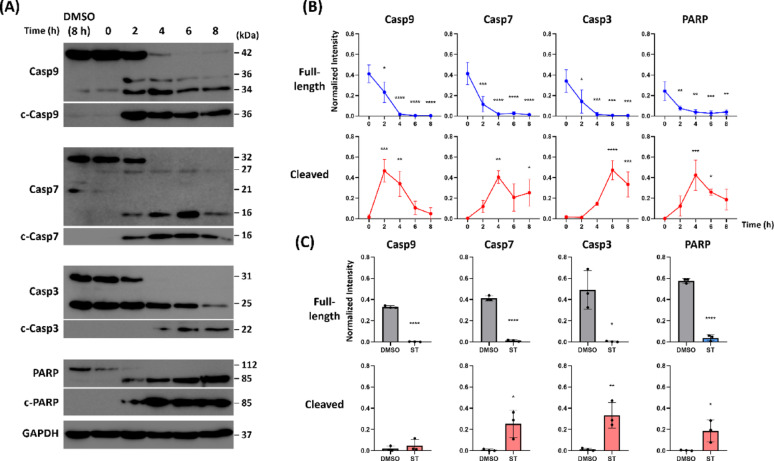
SP strategy in apoptosis study. HeLa cells were treated with 1 µM staurosporine to induce apoptosis. The whole cell lysates obtained at 2-hour interval were electrophoresed and transferred to NC membranes. Antibodies diluted at 1:1000 were applied onto the membranes using SP strategy without agitation, for 15 min at room temperature. (**A**) Representative Western blot image showing caspase and PARP cleavage. (**B**) Time-dependent expression pattern of full-length and cleaved proteins. (**C**) Comparison of DMSO and 1µM staurosporine (ST) groups at 8 h post-treatment

The antibody stock was diluted at 1:1,000 in TBST with 5% skim milk to prepare a working antibody solution. Using SP strategy, 70 µL antibody solution was applied onto the NC. After 15-min incubation at RT without agitation, the rest of the assay was performed in the usual way. The antibodies successfully detected the expression and the cleavage of caspase-9, −7, and − 3 proteins and the poly(ADP-ribose) polymerase (PARP), marking the time-dependent steps of apoptosis event (Fig. 5A). Staurosporine treatment at 1 µM resulted in an immediate decrease in the expression of the full-length caspase and PARP proteins. The full-length protein levels at 2–8 h post-treatment differed significantly from the 0-hour group (Fig. 5B, ordinary one-way ANOVA with Dunnett’s multiple comparison test, *n* = 3). The cleaved forms exhibited a bell-shaped pattern, peaking between 2 and 6 h post-treatment. Their levels also differed significantly over time (Fig. 5B). At 8 h post-treatment, full-length protein levels were significantly lower than those in the DMSO control (8 h), while cleaved forms were significantly higher (Fig. 5C, unpaired two-tailed t-test, *n* = 3). The only exception was cleaved caspase-9, which returned to the basal level after reaching its peak at 2 h post-treatment [13]. These results indicate that SP strategy could be used in researches that require semi-quantitative analysis of proteins. Also, various antibodies at a common working concentration (1:1,000) could be successfully used for SP strategy.

## Discussion

The SP strategy outlined in this study helps conserve the antibodies. Specifically, the flexibility in adjusting the volume of antibody according to the size of each membrane is a clear advantage of SP strategy, whereas CV method uses a fixed volume regardless of membrane dimensions. If a cropped membrane is probed, we could further reduce the antibody solution to apply. We also found that the space formed within SP itself serves as a small humidifying chamber. SP is typically made of polypropylene (PP), which naturally tends to stick to itself due to electrostatic charge [14] and Van der Waals force [15, 16]. These characteristics are advantageous in trapping the liquid and the vapor inside the SP unit without additional sealing, preventing the small volume solution from evaporating during incubation.

When SP leaflets form a thin film of antibody solution, the downward pressure exerted by the upper leaflet is given as $\:{P}_{w}=W/A$, where $\:W$ is the weight of the leaflet and $\:A$ is the contact area between the liquid and the leaflet’s surface. The opposing capillary pressure, $\:\varDelta\:{P}_{c}$, is described by the Young-Laplace equation, $\:\varDelta\:{P}_{c}=\gamma\:\left(1/{R}_{1}+1/{R}_{2}\right)$, where $\:\gamma\:$ is the surface tension coefficient, and $\:{R}_{1}$ and $\:{R}_{2}$ are the principal radii of curvature of the liquid surface [17, 18]. It is the weight of the upper leaflet that counteracts the natural curvature caused by surface tension, flattening the liquid surface that would otherwise form a dome. However, if the leaflet is too heavy, the antibody solution may be excessively displaced laterally, increasing the distance that antibody molecules must diffuse.

The SP strategy provides a practical solution for immunodetection by removing the need for agitation. In traditional Western blot assays, the requirement for continuous shaking often introduces logistical constraints. An orbital shaker or rocker is necessary, and as the number of users or the scale of experiments increases, so does the demand for additional equipment. The greatest issue is that 4 °C overnight incubation requires a cold room. In labs without such facilities, researchers resort to using a refrigerator which is accompanied by limited space, difficulties for power supply, and the risk of mechanical friction and rust. In contrast, SP enables an agitation-free protocol, and it only requires a stable surface to place the SP units. This makes the method extremely practical and accessible for a wide range of laboratory setups.

Furthermore, the SP strategy improves the immunodetection step by facilitating the reaction speed. For an antigen-antibody binding reaction on a solid surface, the overall timescale depends on the characteristic time for reaction at the surface ($\:{\tau\:}_{rxn}$) and the characteristic time for antibodies to diffuse to the reaction site ($\:{\tau\:}_{diff}$) [19]. $\:{\tau\:}_{rxn}$ describes the timescale for an antibody to bind to an antigen at the binding site. When the antibody concentration is significantly larger than that of the antigen, the reaction approximates to a pseudo-first-order reaction [20], where $\:{\tau\:}_{rxn}$ is given as $\:{\tau\:}_{rxn}\:\sim\:1/{k}_{1}\left[Ab\right]$. $\:{k}_{1}$, the association rate constant, ranges 10^5^–10^6^ M^−1^$\:\cdot\:$s^−1^ for an antigen-antibody reaction in general [21, 22]. If we assume $\:{k}_{1}$ to be 5 $\:\times\:$ 10^5^ M^−1^$\:\cdot\:$s^−1^ and set $\:\left[Ab\right]$ at 1 µg/mL, $\:{\tau\:}_{rxn}$ is approximated to 300 s (5 min). $\:{\tau\:}_{diff}$ describes the timescale for the antibody to diffuse across a given diffusion length to reach the antigen. Derived from the Fick’s law, $\:{\tau\:}_{diff}$ is approximated as $\:{\tau\:}_{diff}\:\sim\:{L}^{2}/2D$, where $\:L$ is the diffusion length (the height of the solution) and $\:D$ is the diffusion coefficient of the antibody (4 $\:\times\:$ 10^−7^ cm^2^/s on average) [23]. In the SP strategy method, $\:L$ is estimated to be less than 100 μm, if the porosity and tortuosity of the NC membrane is ignored, producing $\:{\tau\:}_{diff}$ ~ 250 s. These characteristic times indicate a time-scale approximation that helps expect the orders-of-magnitude time required for the overall reaction equilibrium to be completed, where the equilibrium of the reaction is reached after 4–5 $\:{\tau\:}_{total}$. In the SP settings, the total reaction time is expected to be on the order of minutes. This theoretical estimation of the reaction time aligns well with our results in Fig. 4.

However, a boundary layer can form near the membrane surface, where the convective flow becomes minimal, and mass transport is governed primarily by diffusion even if the system is agitated. Inside the membrane, antibodies solely depend on diffusion to locate and bind to the target proteins. This process is very slow due to the large size of antibodies (~ 150 kDa), resulting in a diffusion-limited reaction [10, 20]. Additionally, the tortuous pore network of the NC membrane increases the effective diffusion path beyond the direct Euclidean distance. It was reported that in such porous environments, steric hindrance and hydrodynamic interactions between large molecules and the pore walls can further hinder the diffusion [24]. Therefore, the porous space within the NC membrane imposes a substantial delay on antibody transport and cannot be explained by diffusion or reaction time alone.

We speculate that the blotting of excess liquid plays a crucial role in reducing the incubation time. This step not only prevents dilution of the antibody solution by residual buffer, but also partially removes the liquid occupying the pore spaces of the membrane. When fresh antibody solution is added, capillary infiltration occurs as the solution is drawn into the emptied pore network. During this process, the bulk flow of the solvent actively transports antibody molecules into the membrane. This convective mass transport, driven by capillary forces, is far more efficient than passive diffusion. In other words, removal of liquid from the pore space of the NC membrane facilitates convective delivery of antibodies. Studies using thin film approach have employed a similar principle when they air-dried the PVDF membranes for 10 min before applying the primary antibody [5, 6]. Similarly, a study suggested that completely air-drying a PVDF membrane at 40 °C for 10 min, followed by standard Western blot procedures, could detect sufficient signals by only 25-min incubation with primary antibody [25]. By contrast, there is limited information available regarding drying NC membranes to facilitate the antibody incubation process. Some researchers appear to dry NC membranes after protein transfer, but this practice carries the risk of disrupting the hydration shell essential for preserving protein conformation [26]. Complete drying might induce an irreversible change to the original structure of the protein and compromise the antigenicity. The blotting process in SP strategy is thought to minimize the disruption to the hydration shell surrounding the membrane-bound proteins. Notably, across over 60 antibodies tested with the SP method to date, we have not observed any loss in antibody sensitivity or specificity attributable to the blotting step.

The incubation temperature also contributes to the condition. We performed the incubation at RT, which is expected to accelerate the reaction by increasing both $\:{k}_{1}$ and $\:D$. A study examined temperature-dependent changes in the association rate constant (*k*_1_) for four different antibodies, reporting increases ranging from 1.51-fold to 7.78-fold as the temperature rose from 1 °C to 25 °C [27].

To summarize, this study presents a strategy to significantly reduce both antibody usage and the incubation time in Western blot, as well as removing the need for agitation. The SP method enables semi-quantitative detection with specificity comparable to the CV method, while using minimal antibody volumes tailored to the size of the NC membrane. Because it uses small antibody volumes, the SP method can be performed at RT, without concerns about antibody degradation that often occurs during the antibody reuse. Furthermore, SP strategy does not require agitation, avoiding the logistical constraints associated with equipment like orbital shakers or rockers, or a cold room. Finally, SP method allows effective probing within 15 min at RT. As a result, the entire Western blot assay can be significantly streamlined, reducing the time from sampling to detection (Figure S2).

## Conclusion

The SP strategy provides a universally accessible protocol for Western blot, improving its antibody conservation and incubation time without any expensive, specialized device or consumable. The SP method proposed in this study provides a viable option for performing high-quality Western blotting using only small amounts of antibody. This strategy is not only advantageous for rare antibodies at the laboratory level, but also reduces resource use, promoting environmental sustainability. Furthermore, the ability to obtain Western blot results on the same day enhances research productivity. These advantages could be achieved without any specialized devices or consumables. Therefore, we expect that SP method may serve as a practical protocol for a wide range of researchers across different fields to reduce the usage of antibody and the time for Western blot assays.

## Supplementary Information

Below is the link to the electronic supplementary material.

Supplementary Material 1. **Figure S1.** Schematic illustration of SP strategy. A small volume of antibody is applied on a semi-dried NC membrane and then a SP leaflet is placed on it. The antibody solution is spread over the membrane by capillary action.

Supplementary Material 2. **Video S1.** Demonstration of SP strategy.

Supplementary Material 3. **Table S1.** Examples of minimal antibody volume depending on the number of samples. The vertical length of the membrane was 4.5 cm.

Supplementary Material 4. **Figure S2.** Optimization of the antibody concentration for SP. (A) A representative blot image of GAPDH (10 µg lysate), α-tubulin (20 µg), and β-actin (20 µg) proteins comparing CV and SP. (B) The signal intensity and the antibody concentration (0.1–1.0 µg/mL) in SP strategy showed a positive Pearson’s correlation. (C) The SP antibodies used at 1.0 µg/mL exhibited signal intensities comparable to the CV antibodies used at 0.1 µg/mL.

Supplementary Material 5. **Table S2.** Pearson correlation parameters across the antibody concentrations (0.1, 0.2, 0,5, 1.0 µg/mL).

Supplementary Material 6. **Table S3.** Pearson correlation parameters between the log_10_[lysate] and the signal intensity in CV and SP groups.

Supplementary Material 7. **Table S4.** Pearson correlation parameters between the log_10_[lysate] and the signal intensity in agitated and non-agitated incubation groups.

Supplementary Material 8. **Table S5.** Incubation time-dependent Pearson correlation parameters between the log_10_[lysate] and the signal intensity.

Supplementary Material 9. **Figure S3.** Illustration summarizing advantages of SP strategy compared to CV method.

## Data Availability

No datasets were generated or analysed during the current study.

## Abbreviations

AG: Agitated
CV: Conventional
HRP: Horseradish peroxidase
NA: Non-agitated
NC: Negative control
RPM: Revolutions per minute
RT: Room temperature
SP: Sheet protector
