# Supplementary material for: Sheet Protector Strategy for Western Blot to Reduce Antibody Consumption and Incubation Time
Source: Biol Proced Online. 2025 Sep 24;27:37. doi: 10.1186/s12575-025-00300-6 (PMC12462392; doi:10.1186/s12575-025-00300-6)
Supplement: Supplementary file 1 — Supplementary Material 1. Figure S1. Schematic illustration of SP strategy. A small volume of antibody is applied on a semi-dried NC membrane and then a SP leaflet is placed on it. The antibody solution is spread over the membrane by capillary action. [file 12575_2025_300_MOESM1_ESM.pdf]

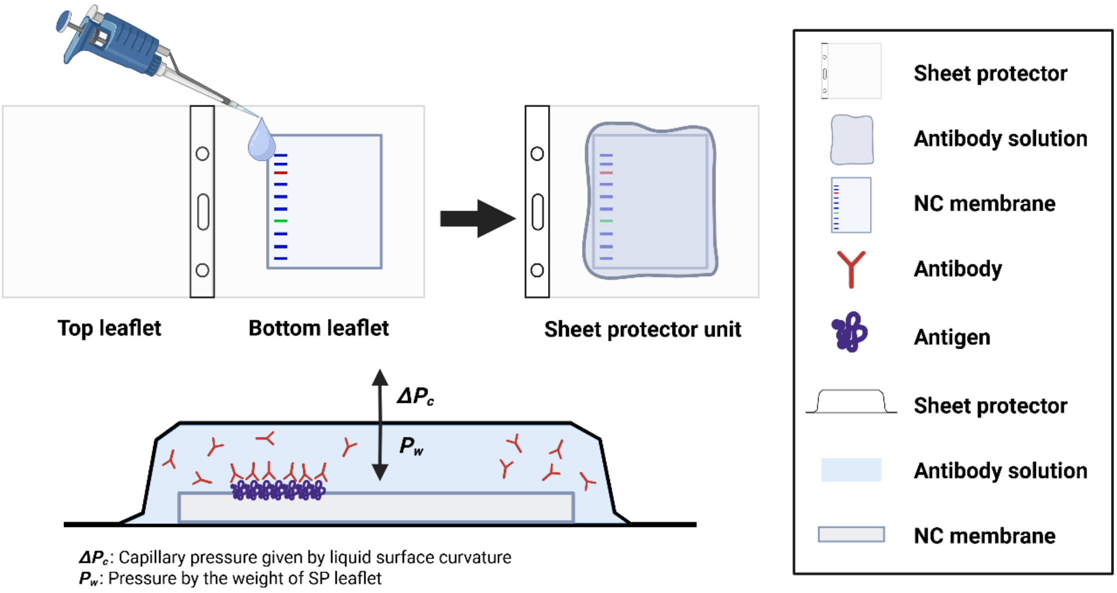

Figure S1. Schematic illustration of SP strategy. A small volume of antibody is applied on a semi-dried NC membrane and then a SP leaflet is placed on it. The antibody solution is spread over the membrane by capillary action.
