# Supplementary material for: Sheet Protector Strategy for Western Blot to Reduce Antibody Consumption and Incubation Time
Source: Biol Proced Online. 2025 Sep 24;27:37. doi: 10.1186/s12575-025-00300-6 (PMC12462392; doi:10.1186/s12575-025-00300-6)
Supplement: Supplementary file 3 — Supplementary Material 3. Table S1. Examples of minimal antibody volume depending on the number of samples. The vertical length of the membrane was 4.5 cm. [file 12575_2025_300_MOESM3_ESM.pdf]

No. of samples	No. of lanes (n)	Width (cm)	Area (cm^2)	V_{cover} (μL)
2	3	1.7	7.65	30
4	5	2.7	12.15	50
9	10	5.2	23.4	100
14	15	7.7	34.65	150

Table S1. Examples of minimal antibody volume depending on the number of samples. The vertical length of the membrane was 4.5 cm.
