# Supplementary material for: Sheet Protector Strategy for Western Blot to Reduce Antibody Consumption and Incubation Time
Source: Biol Proced Online. 2025 Sep 24;27:37. doi: 10.1186/s12575-025-00300-6 (PMC12462392; doi:10.1186/s12575-025-00300-6)
Supplement: Supplementary file 4 — Supplementary Material 4. Figure S2. Optimization of the antibody concentration for SP. (A) A representative blot image of GAPDH (10 µg lysate), α-tubulin (20 µg), and β-actin (20 µg) proteins comparing CV and SP. (B) The signal intensity and the antibody concentration (0.1–1.0 µg/mL) in SP strategy showed a positive Pearson’s correlation. (C) The SP antibodies used at 1.0 µg/mL exhibited signal intensities comparable to the CV antibodies used at 0.1 µg/mL. [file 12575_2025_300_MOESM4_ESM.pdf]

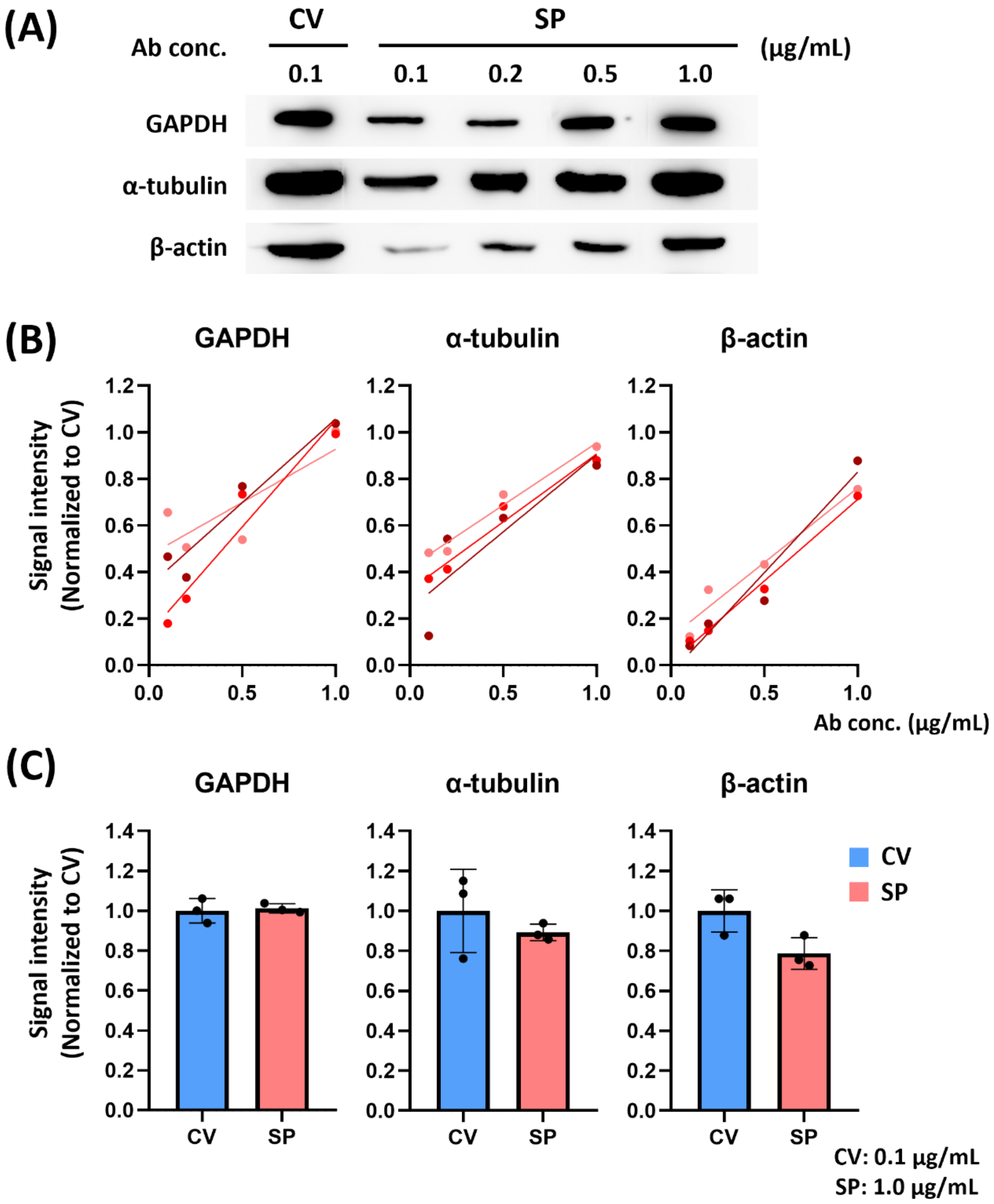

Figure S2. Optimization of the antibody concentration for SP. (A) A representative bl ot image of GAPDH (10 µg lysate), α-tubulin (20 µg), and β-actin (20 µg) proteins c omparing CV and SP. (B) The signal intensity and the antibody concentration (0.1–1.0 µg/mL) in SP strategy showed a positive Pearson's correlation. (C) The SP antibodies used at 1.0 µg/mL exhibited signal intensities comparable to the CV antibodies used a t 0.1 µg/mL
