# Supplementary material for: Sheet Protector Strategy for Western Blot to Reduce Antibody Consumption and Incubation Time
Source: Biol Proced Online. 2025 Sep 24;27:37. doi: 10.1186/s12575-025-00300-6 (PMC12462392; doi:10.1186/s12575-025-00300-6)
Supplement: Supplementary file 5 — Supplementary Material 5. Table S2. Pearson correlation parameters across the antibody concentrations (0.1, 0.2, 0,5, 1.0 µg/mL). [file 12575_2025_300_MOESM5_ESM.pdf]

Group	Replicates	r	R^2	p
**GAPDH**	Set 1	0.8083	0.6533	0.1917
	Set 2	0.9693	0.9395	0.0307
	Set 3	0.9637	0.9286	0.0363
**α-tubulin**	Set 1	0.9863	0.9728	0.0137
	Set 2	0.9821	0.9646	0.0179
	Set 3	0.8726	0.7614	0.1274
**β-actin**	Set 1	0.9771	0.9547	0.0229
	Set 2	0.9959	0.9918	0.0041
	Set 3	0.9743	0.9493	0.0257

Table S2. Pearson correlation parameters across the antibody concentrations (0.1, 0.2, 0,5, 1.0 μg/mL).
