# Supplementary material for: Sheet Protector Strategy for Western Blot to Reduce Antibody Consumption and Incubation Time
Source: Biol Proced Online. 2025 Sep 24;27:37. doi: 10.1186/s12575-025-00300-6 (PMC12462392; doi:10.1186/s12575-025-00300-6)
Supplement: Supplementary file 6 — Supplementary Material 6. Table S3. Pearson correlation parameters between the log10[lysate] and the signal intensity in CV and SP groups. [file 12575_2025_300_MOESM6_ESM.pdf]

Group	Replicates	r	R^2	p
**GAPDH (CV)**	**Set 1**	0.9904	0.9809	0.0001
	**Set 2**	0.9945	0.989	<0.0001
	**Set 3**	0.9631	0.9276	0.002
**GAPDH (SP)**	**Set 1**	0.9832	0.9667	0.0004
	**Set 2**	0.9843	0.9689	0.0004
	**Set 3**	0.9252	0.856	0.0082
**α-tubulin (CV)**	**Set 1**	0.9937	0.9874	0.0006
	**Set 2**	0.9919	0.9839	0.0009
	**Set 3**	0.979	0.9583	0.0037
**α-tubulin (SP)**	**Set 1**	0.9458	0.8945	0.015
	**Set 2**	0.9976	0.9951	0.0001
	**Set 3**	0.9639	0.9292	0.0082
**β-actin (CV)**	**Set 1**	0.8975	0.8056	0.1025
	**Set 2**	0.9689	0.9388	0.1591
	**Set 3**	0.9833	0.9669	0.0167
**β-actin (SP)**	**Set 1**	0.9586	0.9189	0.0414
	**Set 2**	0.9999	0.9999	0.0077
	**Set 3**	0.9964	0.9928	0.0036

Table S3. Pearson correlation parameters between the $\log_{10}$[lysate] and the signal intensity in CV and SP groups.
