# Supplementary material for: Sheet Protector Strategy for Western Blot to Reduce Antibody Consumption and Incubation Time
Source: Biol Proced Online. 2025 Sep 24;27:37. doi: 10.1186/s12575-025-00300-6 (PMC12462392; doi:10.1186/s12575-025-00300-6)
Supplement: Supplementary file 7 — Supplementary Material 7. Table S4. Pearson correlation parameters between the log10[lysate] and the signal intensity in agitated and non-agitated incubation groups. [file 12575_2025_300_MOESM7_ESM.pdf]

Group	Replicates	r	R^2	p
**GAPDH (AG)**	**Set 1**	0.9521	0.9066	0.0034
	**Set 2**	0.9688	0.9385	0.0014
	**Set 3**	0.971	0.9429	0.0012
**GAPDH (NA)**	**Set 1**	0.9611	0.9237	0.0022
	**Set 2**	0.9733	0.9473	0.0011
	**Set 3**	0.9767	0.9539	0.0008
**α-tubulin (AG)**	**Set 1**	0.9702	0.9412	0.0062
	**Set 2**	0.9672	0.9355	0.0071
	**Set 3**	0.9539	0.9099	0.0118
**α-tubulin (NA)**	**Set 1**	0.9644	0.93	0.008
	**Set 2**	0.9705	0.9419	0.006
	**Set 3**	0.9812	0.9627	0.0031
**β-actin (AG)**	**Set 1**	0.9866	0.9733	0.0134
	**Set 2**	0.8906	0.7931	0.1094
	**Set 3**	0.9639	0.9291	0.0361
**β-actin (NA)**	**Set 1**	0.9913	0.9828	0.0087
	**Set 2**	0.961	0.9235	0.039
	**Set 3**	0.9675	0.9361	0.0325

Table S4. Pearson correlation parameters between the log10[lysate] and the signal intensity in agitated and non-agitated incubation groups.
