# Supplementary material for: Sheet Protector Strategy for Western Blot to Reduce Antibody Consumption and Incubation Time
Source: Biol Proced Online. 2025 Sep 24;27:37. doi: 10.1186/s12575-025-00300-6 (PMC12462392; doi:10.1186/s12575-025-00300-6)
Supplement: Supplementary file 8 — Supplementary Material 8. Table S5. Incubation time-dependent Pearson correlation parameters between the log10[lysate] and the signal intensity. [file 12575_2025_300_MOESM8_ESM.pdf]

Group	Replicates	r	R^2	p
**GAPDH (5 min)**	**Set 1**	0.9821	0.9644	0.0179
	**Set 2**	0.9786	0.9578	0.0037
	**Set 3**	0.9932	0.9864	0.0068
**GAPDH (15 min)**	**Set 1**	0.9973	0.9946	0.0002
	**Set 2**	0.9865	0.9731	0.0019
	**Set 3**	0.9681	0.9373	0.0015
**GAPDH (120 min)**	**Set 1**	0.9746	0.9498	0.001
	**Set 2**	0.9874	0.9749	0.0002
	**Set 3**	0.9858	0.9717	0.0003
**GAPDH (O/N, 4°C)**	**Set 1**	0.9989	0.9978	0.0011
	**Set 2**	0.982	0.9643	0.0029
	**Set 3**	0.9711	0.9431	0.0059
**α-tubulin (5 min)**	**Set 1**	0.9806	0.9615	0.0194
	**Set 2**	0.9857	0.9717	0.002
	**Set 3**	0.9797	0.9599	0.0203
**α-tubulin (15 min)**	**Set 1**	0.9964	0.9928	0.0003
	**Set 2**	0.995	0.99	0.0004
	**Set 3**	0.9982	0.9964	0.0001
**α-tubulin (120 min)**	**Set 1**	0.9933	0.9867	0.0007
	**Set 2**	0.9465	0.8958	0.0147
	**Set 3**	0.9509	0.9042	0.013
**α-tubulin (O/N, 4°C)**	**Set 1**	0.9711	0.9429	0.0059
	**Set 2**	0.9583	0.9183	0.0417
	**Set 3**	0.9934	0.9869	0.0006
**β-actin (5 min)**	**Set 1**	0.968	0.937	0.032
	**Set 2**	0.9908	0.9817	0.0092
	**Set 3**	0.9653	0.9318	0.0347
**β-actin (15 min)**	**Set 1**	0.9976	0.9953	0.0024
	**Set 2**	0.9913	0.9828	0.0087
	**Set 3**	0.9977	0.9954	0.0023
**β-actin (120 min)**	**Set 1**	0.9693	0.9396	0.0307
	**Set 2**	0.9903	0.9808	0.0097
	**Set 3**	0.9484	0.8994	0.0516
**β-actin (O/N, 4°C)**	**Set 1**	0.9998	0.9995	0.0141
	**Set 2**	0.9736	0.9479	0.0264
	**Set 3**	0.9719	0.9445	0.0281

Table S5. Incubation time-dependent Pearson correlation parameters between the log_{10}[lysate] and the signal intensity.
