# Supplementary material for: Sheet Protector Strategy for Western Blot to Reduce Antibody Consumption and Incubation Time
Source: Biol Proced Online. 2025 Sep 24;27:37. doi: 10.1186/s12575-025-00300-6 (PMC12462392; doi:10.1186/s12575-025-00300-6)
Supplement: Supplementary file 9 — Supplementary Material 9. Figure S3. Illustration summarizing advantages of SP strategy compared to CV method. [file 12575_2025_300_MOESM9_ESM.pdf]

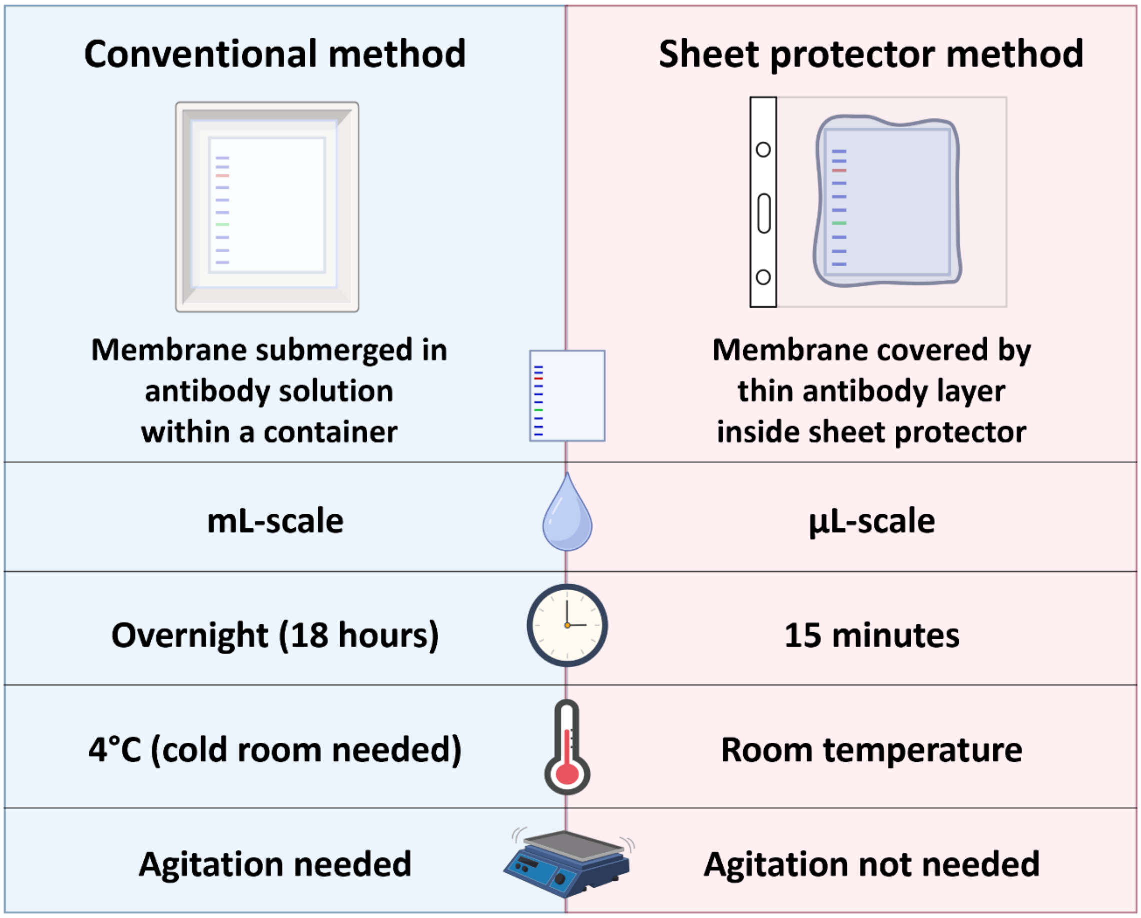

Figure S3. Illustration summarizing advantages of SP strategy compared to CV method.
